# Metabolomics for Preclinical Detection of Diabetic Kidney Disease: A Comprehensive Review

**DOI:** 10.3390/ijms27020998

**Published:** 2026-01-19

**Authors:** Michael Garoufis, Sissy Foteini Sakkou, Christina E. Kostara, Eleni Bairaktari, Vasilios Tsimihodimos

**Affiliations:** 1 Laboratory of Clinical Chemistry, Faculty of Medicine, School of Health Sciences, University of Ioannina, 45110 Ioannina, Greece; michalisgaroufis@gmail.com (M.G.); chkostara@gmail.com (C.E.K.); ebairakt@uoi.gr (E.B.); 2 Department of Internal Medicine, Faculty of Medicine, School of Health Sciences, University of Ioannina, 45110 Ioannina, Greece; sissy_sakkou@hotmail.com

**Keywords:** diabetic kidney disease, diabetes mellitus, metabolomics, predictive biomarkers

## Abstract

Diabetic kidney disease (DKD) affects up to 40% of individuals with diabetes and remains the leading cause of end-stage renal disease worldwide. Current biomarkers, such as albuminuria and estimated glomerular filtration rate, detect disease only after substantial kidney injury, limiting early intervention. Metabolomics offers unique potential to identify early biochemical changes preceding the clinical onset of DKD. This review synthesizes evidence from animal and human studies in diabetes without overt kidney disease, highlighting early perturbations in energy metabolism (TCA cycle, beta-oxidation, glycolysis) as well as alterations in amino acid, nucleotide and urea cycle pathways associated with future DKD risk. We discuss methodological considerations, translational relevance, and current research gaps and outline strategies for integrating metabolomics into predictive diagnostics. Early, non-invasive metabolic biomarkers may enable more precise risk stratification and timely intervention to improve patient outcomes.

## 1. Introduction

Diabetes mellitus (DM) represents a rapidly escalating crisis in global public health. The condition currently impacts approximately 589 million people aged 20–79. Driven by aging demographics and the prevalence of Western lifestyle, this number is anticipated to rise by 45% over the next 25 years, potentially affecting 852.5 million people by 2050. The burden of disease disproportionately affects older adults, where diabetes is a contributing factor in 9.2% of all-cause mortality and is responsible for 63% diabetes-related deaths [1].

Among the complications associated with diabetes, diabetic kidney disease (DKD) is a severe microvascular complication that develops in approximately 20–40% of patients [2]. It is currently the primary driver of end-stage kidney disease (ESKD) globally, accounting for nearly 44% of all cases [3]. Although kidney replacement treatment (KRT) is often necessary for survival in ESKD, its accessibility and financial sustainability remain major issues, particularly in low- and middle-income countries. The number of patients receiving KRT is expected to reach 4.35 million by 2030. Beyond its impact on renal function and the burden that ESKD holds, DKD substantially increases cardiovascular disease (CVD) risk leading to a 16-year loss in life expectancy compared to the general population [2,3].

The Kidney Disease: Improving Global Outcomes (KDIGO) framework defines chronic kidney disease (CKD) based on structural or functional kidney abnormalities, persisting for at least 3 months with potential health implications. Diagnosis requires either a sustained reduction in estimated glomerular filtration rate (eGFR) below 60 mL/min/1.73 m^2^ or evidence of albuminuria, typically defined as a urine albumin-to-creatinine ratio (UACR) > 30 mg/g in a random spot urine sample or persistent albuminuria (>300 mg/24 h) over a period of three months or more, irrespective of eGFR [4]. DKD is diagnosed clinically based on the presence of CKD in the absence of other primary kidney diseases [2].

However, both albuminuria and reduced eGFR occur after the establishment of diabetes-driven histopathological changes as suggested by autopsy data from individuals with diabetes [5]. Furthermore, DKD progression is widely heterogeneous: some patients experience rapid kidney function decline in the absence of severe albuminuria, whereas others show regression of albuminuria over time [5,6]. This variability complicates risk stratification based solely on traditional biomarkers.

Given that early diagnosis and timely intervention remain the most effective strategies to prevent progression and adverse outcomes in DKD there is a critical need for novel, sensitive and noninvasive biomarkers capable of detecting disease before overt clinical manifestations [7].

Metabolomics, the comprehensive profiling of small-molecule metabolites in biological samples that offers a promising avenue to address this need [8,9]. Because metabolites reflect both genetic and environmental influences, metabolomic data provide information complementary to that obtained from genomics and/or proteomics. Recent advances have enabled the use of metabolomics to elucidate complex biochemical pathways and identify early metabolic alterations in multifactorial, metabolic diseases such as DKD [8].

While several studies have summarized the general metabolomic features associated with DKD, most have focused on cross-sectional or late-stage disease cohorts [8,10,11,12]. To date, no comprehensive review has specifically synthesized findings from studies that evaluate the predictive utility of metabolomic biomarkers in individuals with diabetes but without clinically overt kidney disease. Given the pressing need for early, noninvasive and predictive tools to identify individuals at highest risk for progression, this review aims to fill that gap.

We summarize recent findings from both animal models and human longitudinal studies that have applied metabolomics to uncover early biochemical alterations preceding DKD onset. We highlight recurrent metabolic pathways implicated in disease initiation, evaluate the performance of proposed biomarkers and discuss translational challenges and opportunities for incorporating metabolomics-derived biomarkers into risk stratification and clinical-decision making.

## 2. Diabetic Kidney Disease: From Clinical Definition to Unmet Diagnostic Needs

### 2.1. Definition

Diabetic kidney disease is one of the most common and serious complications of both T1DM and T2DM. Clinically, it is defined as the presence of CKD in individuals with diabetes mellitus, in the absence of signs suggesting alternative primary renal disease [13,14]. According to the 2022 KDIGO guidelines, CKD is diagnosed when either a persistent reduction in estimated glomerular filtration rate [(eGFR) < 60 mL/min/1.73 m^2^] and/or elevated urinary albumin excretion [albumin-to-creatinine ratio (ACR) ≥ 30 mg/g] is present for more than three months [4].

The classical progression of DKD was first described by Mogensen in 1985 as a continuum from normoalbuminuria to microalbuminuria (30–300 mg/24 h) in patients with diabetes mellitus [15]. The disease followed a progressive nature with increases in the amount of the excreted albumin reaching >300 mg/24 h, called macroalbuminuria or overt nephropathy. This is followed by a decline in renal function and finally end-stage renal disease. However, since then, this model has evolved. It is now recognized that a substantial proportion of patients with diabetes, particularly those with T2DM, may experience declines in eGFR in the absence of albuminuria, underscoring the heterogeneity of DKD and the involvement of diverse pathophysiological mechanisms [13].

DKD can be considered as an “umbrella” term, encompassing cases in which renal injury is attributed to hyperglycemia per se, comorbidities such as hypertension and obesity, mixed forms and in some cases in other renal diseases. While kidney biopsy remains the gold standard for definitive diagnosis of diabetic nephropathy, it is not routinely performed in clinical practice and is typically reserved for atypical presentations. Consequently, studies based on histopathological findings may overestimate the prevalence of non-diabetic renal disease in this population [16].

### 2.2. Epidemiology

Worldwide 700 million people suffer from chronic kidney disease, of whom almost four million patients require KRT. Currently, CKD is the third fastest-growing cause of death globally and is projected to become the fifth leading cause of years of life lost by 2040 [17]. The true prevalence of the disease is most likely underestimated due to insufficient early detection and screening programs, as well as low public and clinical awareness, particularly in the asymptomatic early stages of the disease.

Kidney disease is associated with high morbidity and mortality, even in the early stages. Over the past three decades, CKD has risen to become the eighteenth leading global cause of disability with an absolute increase of 62% in age-standardized disability adjusted life years (DALYs). Moreover, it ranks as the seventh leading risk factor for death and imposes a substantial economic burden on healthcare systems due to its complex management demands [17].

Diabetes mellitus is the leading cause of CKD globally. DKD affects approximately 50% of individuals with T2DM and 30% of those with T1DM. However, these estimates are imprecise due to the frequent co-occurrence of other kidney pathologies, particularly in T2DM, and the limited use of kidney biopsy, which restricts definitive etiological classification [14].

Increased urinary albumin excretion remains the most common clinical manifestation of DKD. Albuminuria develops at an annual incidence of 8% in T2DM and 2–3% in T1DM, while the incidence of eGFR decline appears comparable across diabetes types, ranging between 2 and 4% per year. Importantly, awareness of CKD among diabetes patients remains low, rising from just 3% in those with preserved kidney function (eGFR > 90 mL/min/1.73 m^2^) to 53% in those with stage G4 [14].

### 2.3. Pathophysiology

The pathophysiology of diabetic kidney disease is multifactorial, primarily driven by three interconnected axes: metabolic, hemodynamic, and inflammatory processes [18]. Chronic hyperglycemia is the principal etiological factor, contributing simultaneously to all three pathways. It induces metabolic derangements and hemodynamic alterations that promote endothelial dysfunction, glomerular hyperfiltration, and chronic inflammation [5,18,19].

Clinically and mechanistically, the disease trajectory begins with a phase of glomerular hyperfiltration [20,21]. This phenomenon is driven by a maladaptive feedback loop between the glomerulus and the proximal tubule. In the setting of diabetes, the kidneys undergo significant hypertrophy, largely due to the enlargement of proximal tubular cells caused by cytokines and growth factors in response to hyperglycemia. To manage the increased filtered load driven by systemic hyperglycemia, proximal tubular cells upregulate the expression of sodium-glucose cotransporters and sodium-hydrogen exchangers leading to enhanced reabsorption of sodium and glucose [22]. This reduction in distal sodium delivery is sensed by the macula densa, which via tubuloglomerular feedback triggers the dilation of the afferent arteriole. Concurrently, an imbalance in vasoactive humoral factors increases post-glomerular resistance, leading to elevated intraglomerular pressure and flow. This hemodynamic burden imposes a severe bioenergetic cost. Hyperfiltration forces the renal tubules to operate at maximum capacity, increasing their oxygen and ATP consumption [23].

Importantly, glucose uptake in microvascular endothelial cells occurs independently of insulin and is solely dependent on ambient glucose concentrations. In the hyperglycemic state, excessive intracellular glucose accumulation in glomerular and tubular cells diverts metabolism towards alternative, non-glycolytic pathways such as the polyol (aldose reductase) pathway and the hexosamine biosynthetic pathway. These metabolic shifts enhance the generation of advanced glycation end products (AGEs), which in turn lead to endothelial dysfunction, pro-inflammatory signaling cascades and oxidative stress [19,24]. Mitochondrial dysfunction and NADPH oxidase activation are the main sources of reactive oxygen species (ROS) production, creating a vicious cycle of redox imbalance and inflammation [25,26].

Persistent oxidative stress is closely linked to hypoxia and mitochondrial dysfunction, key contributors to tubulointerstitial injury. In the diabetic kidney, an imbalance between oxygen supply and demand results in hypoxia, particularly in proximal tubular cells. Furthermore, proximal tubular cells which are highly reliant on oxidative phosphorylation for energy production, undergo mitochondrial dysfunction under hyperglycemic and hypoxic conditions. This impairs ATP generation, increases mitochondrial ROS production and reinforces oxidative damage [27]. From a therapeutic perspective of view, the relevance of these mechanisms is validated by the efficacy of novel agents such as SGLT2 inhibitors and GLP-1 receptor agonists. By reversing hemodynamic overload and restoring metabolic homeostasis, these agents have been shown to preserve mitochondrial integrity and mitigate the bioenergetic burden on the diabetic kidney [28,29].

Hypertension, frequently coexisting with diabetes, further exacerbates renal injury and plays a significant role in both the initiation and progression of DKD [19]. Local activation of the renin–angiotensin–aldosterone system (RAAS) plays a central role in DKD pathogenesis. Angiotensin II not only induces intraglomerular hypertension via its potent vasoconstrictive effects but also promotes oxidative stress, primarily through superoxide production by NADPH oxidase. Additionally, it drives mesangial cell hypertrophy and hyperplasia, stimulates extracellular matrix accumulation (e.g., collagen type I), and enhances the expression of profibrotic mediators such as TGF-beta [30,31,32].

The resulting renal damage in DKD encompasses structural and functional alterations, including glomerular hypertrophy and hyperfiltration, as well as glomerular and tubulointerstitial inflammation. These changes are mediated by the accumulation of pro-inflammatory cytokines, chemokines, and profibrotic factors, along with dysregulation of apoptosis and extracellular matrix remodeling. Over time, these pathological processes culminate in thickening of the glomerular basement membrane, podocyte loss, mesangial expansion, tubular atrophy, interstitial fibrosis, and ultimately, glomerulosclerosis [33].

### 2.4. Diagnostic Limitations of Conventional DKD Biomarkers

Diagnosis and staging of DKD are based on eGFR and/or urinary albumin excretion. To date, more than seventy equations have been developed to calculate eGFR, using endogenous filtration biomarkers, most often serum creatinine [34,35]. Of these, CKD-EPI and MDRD are the most commonly used in everyday clinical practice, with similar overall diagnostic accuracy [36]. The continuous revision and development of eGFR calculation models highlight the intrinsic limitations of current biomarkers, implying that the core issue lies not within the equations but within the biological inputs themselves.

An ideal biomarker of kidney function should be freely filtered by the glomerulus, not bound to plasma proteins, and not be reabsorbed, secreted, or metabolized by renal tubular cells. It should be inert, produced at a constant rate and eliminated exclusively via the urine [34]. Creatinine is the product of creatine and phosphocreatine catabolism, mainly in skeletal muscle [35]. Even though creatinine is not protein-bound and is freely filtered by the glomerulus, its renal handling is complex. Under physiological conditions, active tubular secretion accounts for about 10% of total urinary creatinine excretion. Crucially, this percentage rises linearly with GFR decline and in advanced CKD stages can account for a substantial majority of excretion, blunting the rise in serum creatinine and masking the true extent of GFR reduction. On the contrary, under physiological conditions, creatinine reabsorption in the tubules is not observed. However, evidence from limited data suggests that reabsorption may occur under conditions of diminished urinary flow rates, potentially reducing clearance by less than 10% [34,37]. Moreover, its production depends on the subject’s dietary habits, with meat being its major source, and on muscle turnover. As a result, GFR is overestimated by limited protein intake and decreased muscle mass and underestimated by increased protein intake and conditions such as intensive exercise, chronic glucocorticoid use and hyperthyroidism. Apart from urinary excretion, creatinine can be recycled in creatine or creatine be degraded into other products, other than creatinine. Extrarenal creatinine clearance increases in kidney dysfunction, contributing to eGFR overestimation in advanced CKD stages [38]. Creatinine levels are also influenced by biological confounders such as age, sex and race [39]. The widely used Jaffé method is affected by analytical interferences from non-specific chromogens, including glucose, ketones, proteins and other reducing substances, thereby compromising its specificity. Although enzymatic assays offer greater analytical accuracy, they are costly and susceptible to interference from hemolysis, hyperbilirubinemia and monoclonal proteins [34,40]. Moreover, in DKD, estimation equations may either under- or overestimate true GFR due to compensatory adaptations in the remaining nephrons. For example, afferent arteriolar vasodilation can increase single-nephron GFR, even in the absence of overt nephron loss, thereby masking early functional decline [18,41]. As a result of these limitations, the relationship between serum creatinine and measured GFR is not linear but curvilinear. For any given serum creatinine concentration, measured GFR values can vary widely among individuals, reflecting the influence of both biological and analytical variability. This non-linearity limits the sensitivity of serum creatinine for detecting early declines in GFR and contributes to diagnostic imprecision [34].

To overcome the limitations of creatinine use, another serum biomarker, cystatin C has been used to evaluate kidney function. Cystatin C is an endogenous cysteine protease inhibitor synthesized by all nucleated cells at a relatively constant rate. Due to its low molecular weight and positive charge, it is freely filtered by the glomeruli. Unlike creatinine, cystatin C is neither secreted by renal tubular cells nor returned to circulation; rather, it is almost entirely reabsorbed and catabolized by proximal tubular cells, preventing its reappearance in urine under normal physiological conditions. These favorable kinetic properties have prompted the development of more than 15 equations for estimating glomerular filtration rate based on cystatin C since its introduction in 1985. Several of eGFR equations incorporate both cystatin C and serum creatinine to improve diagnostic accuracy [18,34]. However, the clinical utility of cystatin C as a filtration marker is limited by its non-GFR determinants. Its serum concentration exhibits a non-linear, curvilinear relationship with measured GFR, and elevations may occur independently of renal function. Specifically, increased serum cystatin C levels have been documented in individuals with morbid obesity, type 2 diabetes mellitus, hypertension, metabolic syndrome, and in those receiving high-dose glucocorticoid therapy. Additionally, liver dysfunction has been shown to affect cystatin C levels through GFR-independent mechanisms. These confounding factors must be considered when interpreting cystatin C–based eGFR estimates, particularly in populations with complex comorbidities [34,39].

Increased albumin excretion in the urine is the second cornerstone of the diagnosis and staging of CKD. Albuminuria serves as a surrogate marker of endothelial dysfunction and is independently associated with increased cardiovascular mortality and morbidity even in non-diabetic individuals [13]. However, in the context of DKD, albuminuria presents several limitations as a diagnostic and prognostic biomarker. Primarily, albuminuria is a late and non-specific indicator of renal injury in diabetes. While albuminuria reflects compromised glomerular integrity, it may fail to detect early tubulointerstitial pathology. Evidence indicates that tubular injury, as manifested by the excretion of tubular proteins such as *N*-acetyl-β-D-glucosaminidase (NAG), neutrophil gelatinase-associated lipocalin (NGAL) and heart fatty acid-binding protein (H-FABP), can occur in normoalbuminuric patients, suggesting that tubular damage may arise independently of, or even precede, clinically detectable glomerular injury [42]. Consequently, reliance on albuminuria alone may obscure the detection of this early tubulointerstitial damage. Histopathological changes in the diabetic kidney often precede detectable increases in urinary albumin levels. Moreover, elevated urine levels are noticed in most glomerulopathies as well as vigorous exercise, infections, hypertension and heart failure. These influences, coupled with intrinsic biological variability of more than 20% diminish the reliability of a single measurement [5,13,43]. Given this variability, transitions between albuminuria categories should not be interpreted as definitive evidence of disease progression or regression. Instead, such changes warrant cautious interpretation within the broader clinical context. To enhance diagnostic confidence and evaluate the clinical significance of these changes, repeat testing over a 3- to 6-month interval is recommended. Additionally, albuminuria exhibits significant diurnal and intra-individual variability, further complicating its interpretation [43]. Analytical inconsistencies, such as non-standardized assay methods, differences in sampling approaches (24 h urine collections versus spot urine samples) and variability in reporting units, introduce further challenges in clinical decision-making [13]. Emerging recognition that albuminuria may not reliably track disease progression in DKD occurs from data of studies that have identified subsets of patients with DKD who follow non-albuminuric pathways or demonstrate regression of albuminuria over time. These findings underscore a growing dissociation between albuminuria and GFR in diabetes, suggesting that reliance on albuminuria alone fails to capture the full spectrum of renal decline [5,8].

## 3. Metabolomics: Concepts, Technologies and Analytical Framework

### 3.1. Definition and Scope of Metabolomics

Metabolomics, the systematic analysis of the metabolome, which is the complete set of metabolites in a biological system, provides an effective method for the discovery of key, non-invasive diagnostic biomarkers for complex diseases with profound metabolic alterations, such as DKD [44,45].

Metabolites are organic, chemically diverse compounds of low molecular weight (<1500 Da) that serve as reactants, intermediates, or end-products of biochemical reactions and are implicated in multiple signaling pathways as well as in the regulation of cellular functions [45]. Metabolites present with a diversity in size, structure, and polarity and have a wide and dynamic concentration range, making their identification and quantification an analytical challenge [46].

Compared to other “omics’’ approaches, metabolomics offers some advantages. Metabolites are downstream of the processes of transcription and translation, representing the interaction between genes, proteins, individual and external factors. Consequently, metabolomics offers a view that is closer to the phenotype of disease, identifying changes in metabolic pathways that have occurred [8,45,47]. Hence, identified metabolites provide a unique pattern comparable to a fingerprint, serve as direct signatures of biochemical activity, and are easier to correlate with disease phenotype [48,49].

### 3.2. Metabolomics Workflow Overview

The workflow of a metabolomics study is multi-step and consists of experimental design, the pre-analytic treatment of the sample, separation and detection of metabolites, data processing and analysis, statistical analysis and the interpretation of the results [46]. The integrity of the study’s outcome is highly dependent on the quality, consistency, execution and reliability of each step and several procedural check points and controls are required to ensure that the data obtained are reproducible, precise and accurate [45]. Confounding factors need to be carefully addressed during any metabolomic study as differences in body mass index, diabetes and fasting status at sample collection are essential [50].

During the design of the experiment, it is decided whether a targeted or untargeted approach will be followed. In targeted analysis, a predetermined set of metabolites is investigated, typically focusing on one or more related pathways of interest [46,48]. This approach is hypothesis-driven and allows high sensitivity, accurate detection and quantification of a low number of metabolites at any given time.

In contrast, untargeted analysis is hypothesis-generating and aims to simultaneously detect all measurable metabolites in a biological specimen and identify them using software tools based on known or predicted spectral patterns. Even if untargeted metabolomics allows the detection of many metabolites in a single run, high-quality precision is limited whereas the time required for accurate metabolite identification and quantification can be substantial. Untargeted metabolomics is especially useful for the detection of potential biomarkers, followed by a targeted approach to confirm results [46].

### 3.3. Analytical Platforms in Metabolomics

The most often used analytical approaches in metabolomics are nuclear magnetic resonance (NMR) spectroscopy and mass spectrometry (MS) coupled to a chromatographic technique such as gas chromatography (GC) or liquid chromatography (LC), to achieve separation and reduce sample complexity before mass analysis [10,46]. Due to the physicochemical diversity of the metabolites, a single analytical method cannot capture the entire metabolome present in a biological sample [45].

MS-based methods offer superior sensitivity, but different chromatographic conditions are better suited for different kinds of metabolites, making the use of more than one chromatography-MS combination necessary. After chromatography, metabolites enter the MS and undergo ionization while MS resolves metabolites based on their mass-to-charge ratios [10]. In GC-MS analysis, samples need to be thermally stable and volatile, as separation by GC occurs at high temperatures. Chemical derivatization before analysis is needed for samples to be volatile; however, it can result in metabolite loss and complicate analysis because of incomplete derivatization or artifact formation. Its advantages are high sensitivity, specificity and reproducibility as well as lower instrumental-based variability among results compared to LC-MS. In LC-MS analysis, there is no need for sample derivatization and a greater coverage of mass ranges. LC-MS is versatile as several mass analyzers can be coupled to LC, and allows the separation and detection of many different classes of metabolites. Sample ionization needs to occur, after which the mass of the analyte is determined by the mass analyzer as mass-to-charge ratio (*m*/*z*) [46].

NMR spectroscopy uses the magnetic properties of atomic nuclei (e.g., 1H) to determine the structure and abundance of metabolites in a biological specimen. These atomic nuclei have a characteristic spin and when placed in a magnetic field, absorb radiation and resonate at a specific frequency. It is highly reproducible and compared to MS-based methods, NMR spectroscopy requires minimal sample preparation, is not destructive, without the need for an up-front separation or derivatization method, and it can provide absolute quantitation. However, its sensitivity is limited to the detection of relatively abundant analytes, as concentrations can be detected into the micromolar range [10,45,46].

### 3.4. Statistical and Computational Analysis

Statistical analysis is essential in metabolomics for identifying significant differences in metabolite profiles between disease and control groups, ensuring data validity, and presenting results in an interpretable format, particularly for interdisciplinary audiences. Given the high-dimensional and complex nature of metabolomic datasets, multivariate data analysis (MDA) is commonly employed to extract meaningful biological insights [51].

A typical MDA pipeline begins with unsupervised methods, primarily Principal Component Analysis (PCA), which reduces data dimensionality by transforming original variables into a smaller set of orthogonal components (principal components). PCA enables visualization of patterns, clustering, and detection of outliers without prior knowledge of group labels. Subsequently, supervised methods are applied to enhance group discrimination. Partial Least Squares Discriminant Analysis (PLS-DA) and Orthogonal PLS-DA (OPLS-DA) are widely used for this purpose. PLS-DA models the relationship between predictor variables (e.g., metabolite intensities) and a response variable (e.g., disease status), while OPLS-DA further separates predictive and non-predictive (orthogonal) variation, improving interpretability and robustness of the model.

Model performance is typically assessed via cross-validation, using parameters such as R^2^ (explained variance) and Q^2^ (predictive ability). An R^2^ value approaching 1 indicates excellent model fit, while a Q^2^ > 0.5 is considered good, and >0.9 excellent. A difference between R^2^ and Q^2^ (R^2^ − Q^2^) of less than 0.3 suggests that the model is not overfitted. Additionally, permutation tests are conducted to validate model reliability, with statistical significance indicated by *p* < 0.05 [52].

Beyond statistical classification, metabolic pathway analysis enhances biological interpretation by contextualizing differential metabolites within metabolic networks. Given that metabolites often participate in multiple pathways, visualization tools and network-based analyses facilitate the identification of perturbed biochemical routes associated with disease states [39,53].

## 4. Metabolomics in DKD

The close physiological interplay between renal function and systemic metabolism makes DKD an especially compelling condition to study through the lens of metabolomics. The kidneys are not only highly metabolic organs—engaged in extensive energy-demanding processes such as active solute transport and ammonia production—but also serve as key regulators of the circulating metabolome. They influence metabolite concentrations through glomerular filtration, tubular secretion, reabsorption, and enzymatic catabolism [8,10,54]. In parallel, diabetes mellitus, as a systemic metabolic disorder, induces widespread alterations in circulating metabolites, many of which may act as mediators or markers of downstream complications, including DKD. This bidirectional interaction between metabolic dysregulation and renal physiology underscores the potential of metabolomics to both elucidate the pathophysiological mechanisms linking DM to kidney injury and identify novel biomarkers for early detection and risk stratification.

**Figure 1:**
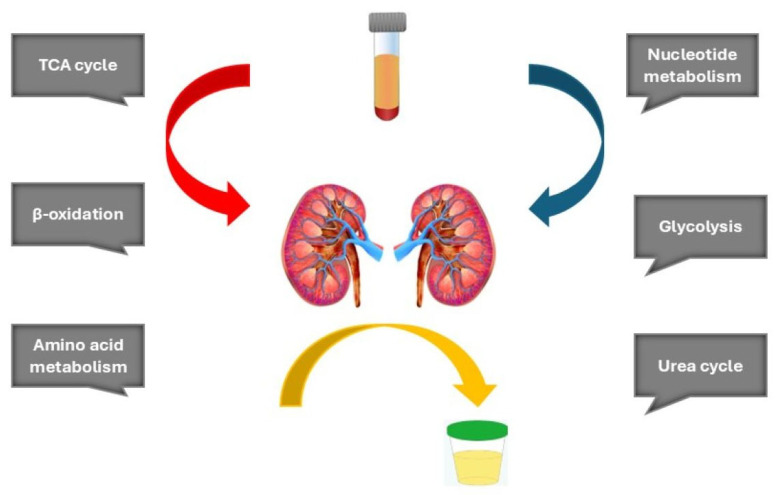
Key metabolic pathways altered early in diabetic kidney disease.

To date, most metabolomic investigations in DKD have employed untargeted approaches, predominantly using serum and urine samples, which together capture a broad spectrum of systemic and kidney-derived metabolites [8,55]. These studies consistently report alterations in key metabolic pathways, including the tricarboxylic acid (TCA) cycle, glycolysis, amino acid metabolism, lipid oxidation, nucleotide metabolism, and the urea cycle (Figure 1). However, as highlighted in a recent meta-analysis by Roointan et al., the majority of these studies are cross-sectional and conducted in individuals with established renal impairment, limiting their utility for identifying early, predictive metabolic signatures of disease onset [56,57].

The following section focuses specifically on studies that attempt to address this gap by investigating the predictive potential of metabolomic profiling in individuals with diabetes but without overt signs of kidney disease.

### 4.1. Insights from Animal Models: Early Metabolic Perturbations in Diabetic Kidney Disease

Animal models provide a controlled environment to study the early pathophysiological events that precede the clinical onset of diabetic kidney disease. Both type 1 and type 2 diabetes models have been employed to characterize metabolic reprogramming in the kidney and systemic biofluids, often before the manifestation of classical clinical markers such as albuminuria or reduced glomerular filtration rate (Table 1).

In a longitudinal metabolomics study using gas chromatography–time-of-flight mass spectrometry (GC-TOFMS), Li et al. evaluated serum and urine metabolic profiles in db/db mice. Prior to the onset of albuminuria (week 10) and as early as week 6, diabetic mice exhibited significantly elevated serum levels of TCA cycle intermediates including fumarate, succinate, citrate, 2-ketoglutarate, and malate, as well as saturated free fatty acids and reduced lysine concentrations. Urinary excretion of citrate, malate, and 3-hydroxybutyrate was also increased, suggesting an upregulated TCA cycle and energy metabolism early in disease development [58].

The integration of multi-omics approaches has further enhanced our understanding of early DKD pathogenesis. Sas et al. combined kidney cortex metabolomics with transcriptomics in db/db mice and non-diabetic controls. Their findings revealed increased metabolic flux through glycolysis, beta-oxidation, and the TCA cycle, along with transcriptomic evidence of mitochondrial dysfunction and enhanced protein acetylation. These data reinforce the central role of mitochondrial dysregulation in early diabetic nephropathy [59].

Mitochondrial and lipid metabolic alterations were also evident in a non-targeted metabolomics study of renal cortex tissue from a T1DM model by Bergman et al. The diabetic mice demonstrated accumulation of unsaturated, non-esterified fatty acids, monoacylglycerols, short and long chain-length acylcarnitines. These signatures suggest defective beta-oxidation and impaired branched-chain amino acid (BCAA) catabolism [60]. Notably, the increase in non-esterified fatty acids results in the accumulation of re-esterification pathway metabolites, including diacylglycerol [61]. Diacylglycerol binds to the cysteine-rich C1 region of PKC, triggering its activation [62,63]. PKC activation, serves as a key mediator of diabetic complications, through mechanisms involving oxidative stress, inflammation, fibrosis (via TGF-beta), and altered renal hemodynamics [24,64].

Disruption of amino acid metabolism was further supported by findings from Rong et al., who demonstrated increased citrulline and decreased aspartate and arginine levels in the renal cortex of diabetic mice. These changes coincided with renal hypertrophy and glomerular hyperfiltration, hallmarks of early DKD, indicating dysregulation in the urea and nitric oxide cycles [65]. Mechanistically, the depletion of aspartate limits the activity of argininosuccinate synthase, which requires aspartate to convert citrulline to arginine. This impairment is consistent with the upregulation of renal gluconeogenesis in the diabetic state, which increases the demand for glucogenic substrates and drains the aspartate pool available for the urea cycle [66]. The resulting arginine deficiency promotes endothelial nitric oxide synthase uncoupling, shifting enzymatic output from nitric oxide to superoxide anion thereby fueling renal oxidative injury [65].

Carbohydrate metabolism also appears to be significantly perturbed in the diabetic kidney. In an elegant study by Kogot-Levin et al., renal tissue from diabetic mice displayed elevated levels of glucose, sorbitol, galactosamine, glucosamine, and other glucose-derived amines. These changes suggest an impaired capacity of the diabetic kidney to manage and excrete glucose-derived metabolites. Moreover, glucose-derived TCA intermediates, including citrate and succinate, were reduced. Importantly, treatment with the SGLT2 inhibitor dapagliflozin partially normalized these metabolic disturbances, underscoring the therapeutic potential of modulating renal glucose handling [67].

Together, these preclinical findings provide mechanistic evidence that metabolic dysregulation, including mitochondrial dysfunction, altered amino acid and lipid metabolism, and glucose dysregulation precedes overt clinical markers of DKD. These findings offer critical insights that guide the interpretation of human metabolomics data, lay the groundwork for translation efforts in humans and highlight candidate pathways for early biomarker discovery.

**Table 1:** Metabolomics investigations in experimental models of diabetic kidney disease.

Study	Diabetes Model	Sample Type	Analytical Platform	Main Findings- Altered Metabolic Pathways
Li et al., 2012 [58]	db/db mice	Serum and urine	GC-TOFMS	↑ serum and urine TCA cycle intermediates four weeks before the onset of albuminuria.
Sas et al., 2016 [59]	BKS db/db mice	Renal cortex	LC/MS-GC/MS	Combined metabolomics and transcriptomics analysis revealed ↑ metabolic flux through glycolysis, beta-oxidation, TCA cycle and mitochondrial dysfunction.
Bergman et al., 2019 [60]	Insulinopenic STZ T1DM rats	Renal cortex	nano-DESI MSI	Accumulation of unsaturated NEFAs, monoacylglycerols and acylcarnitines reflecting defective beta-oxidation and BCAA catabolism.
Rong et al., 2022 [65]	STZ T1DM mice and db/db mice	Renal cortex	UPLC-MS	↑ citrulline, ↓ aspartate and arginine reveal dysregulation in the urea and NO cycles.
Kogot-Levin et al., 2023 [67]	Insulin-deficient Akita mice	Renal cortex	LC/MS	↑ glucose-derived amines and carbohydrates, ↓ glucose-derived TCA intermediates suggesting impaired glucose handling.

↑ increased concentration, ↓ decreased concentration.

### 4.2. Predictive Metabolomics in Human Studies: Identifying Early Biomarkers of DKD

While animal models have provided foundational insights, translational data remains scarce. Only a limited number of prospective metabolomics studies have characterized the early metabolic perturbations predicting incident DKD in humans with diabetes and preserved renal function (Table 2).

In a seminal study nested within the FinnDiane cohort, van der Kloet et al. applied GC-MS and LC-MS to baseline urine samples from 52 normoalbuminuric individuals with T1DM, followed for an average of 5.5 years. Metabolomic profiling identified discriminatory features—including acylglycines (e.g., salicyluric acid, hippuric acid, 2-phenylacetoxy-propionyl-glycine and 3-methylcrotonylglycine), acylcarnitines, and tryptophan metabolites (e.g., tryptophan, indoleacetic acid, kynurenic acid)—that differentiated progressors to micro- or macroalbuminuria from non-progressors, with a multivariate model yielding 75% accuracy and 73% precision [68].

By contrast, Pena et al., using a similar untargeted urine and serum metabolomics approach in a case–control study of 90 individuals with T2DM, failed to identify metabolites predictive of progression to microalbuminuria. This discrepancy may reflect differences in patient characteristics, disease type, or analytical methods [69].

Solini et al. conducted a longitudinal study in T2DM patients (median eGFR: 85 ± 20 mL/min/1.73 m^2^), integrating GC-MS and UPLC-MS/MS data from serum and urine samples. After a median follow-up of 13 years, a composite serum metabolite score (C-glycosyl tryptophan, pseudouridine, and N-acetylthreonine) improved prediction of renal decline (eGFR < 60 mL/min/1.73 m^2^ or ACR ≥ 30 mg/g), increasing the ROC AUC from 0.671 (clinical parameters alone) to 0.739. Urinary metabolites, however, did not significantly enhance predictive performance [70].

In a subgroup analysis of the Family Investigation of Nephropathy and Diabetes (FIND) study, Sas et al. examined baseline urinary metabolomic profiles in T2DM patients without clinical evidence of kidney disease. After a 5-year follow-up, those who progressed to renal dysfunction (eGFR < 65 mL/min/1.73 m^2^ and creatinine > 1.2 mg/dL) exhibited significantly elevated levels of tricarboxylic acid cycle intermediates compared to non-progressors. These findings point to early dysregulation in mitochondrial energy metabolism as a potential mechanism of subclinical kidney injury [59].

Haukka et al. performed untargeted serum metabolomics using UPLC-MS/MS and GC-MS in 200 T1DM individuals with preserved eGFR and normoalbuminuria, followed for a median of 3.2 years. A metabolomic index comprising erythritol, 3-phenylpropionate, and N-trimethyl-S-aminovalerate significantly improved prediction of incident microalbuminuria beyond clinical risk factors (AUC 0.842 vs. 0.797), implicating perturbations in uremic toxin and carnitine metabolism as early harbingers of DKD [71].

In a longitudinal study by Afshinnia et al., 92 individuals with T2DM and iothalamate-based GFR ≥ 90 mL/min at baseline were followed for a median of 9.6 years. Among normoalbuminuric participants, each 1-SD increase in long-chain acylcarnitines (C16–C20) was associated with a 3.36-fold increased risk of DKD progression (defined as ≥40% decline in GFR). Conversely, higher levels of short-chain, low-double bond triacylglycerols (TAGs) conferred a protective effect—emphasizing lipid metabolism as a key determinant of progression risk, even in early disease stages [72].

Mutter et al. used NMR spectroscopy to analyze 2670 urine samples from T1DM individuals in a large observational study. Elevated urinary 2-hydroxyisobutyrate was consistently associated with both overall progression (to higher albuminuria stages or ESKD) and incident albuminuria in the normoalbuminuric subgroup [73].

Two smaller prospective T2DM studies further support the predictive utility of baseline metabolic profiles. Zhu et al. identified serum L-valine and isoleucine as independent predictors of DKD onset and rapid eGFR decline (>3 mL/min/1.73 m^2^ annually) over 69 months [74], while Lin et al. reported that lower phosphatidylcholine 38:4 and higher uric acid levels were associated with new-onset DKD [75].

Finally, in a landmark investigation of the Diabetes Prevention Program Outcomes Study (DPPOS), Perng et al. extended the risk stratification to the pre-diabetic state. Utilizing a cohort of 1947 individuals with prediabetes, the authors analyzed 353 annotated serum metabolites against incident microvascular complications over a 15-year follow-up. Regarding DKD, the study identified metabolic signatures that were largely distinct from those predicting diabetic retinopathy and neuropathy, rather than a common profile shared across all microvascular complications. Specifically, higher baseline levels of histidine were uniquely associated with reduced odds of incident nephropathy, while serine demonstrated a shared protective effect for both nephropathy and neuropathy. Notably, the study also reported treatment-specific interactions, identifying N-carbamoyl-beta-alanine as a significant risk marker for nephropathy specifically within the metformin treatment arm, highlighting the potential of metabolomics to guide personalized therapeutic choices even before the onset of frank diabetes [76].

Collectively, these longitudinal studies consistently converge on metabolic pathways, particularly those related to mitochondrial function (e.g., TCA cycle, beta-oxidation), amino acid metabolism and lipid handling, as early and reproducible harbingers of DKD. Despite differences in analytical methods and populations, the alignment across findings strengthens the case for metabolomics-based risk stratification in individuals with diabetes and preserved kidney function. However, the translation of these discoveries into clinical practice remains unrealized. To date, no single metabolite or multi-marker panel has been adopted for routine risk stratification in the clinical management of DKD.

**Table 2:** Human metabolomics studies predicting DKD before clinical onset.

Study	Follow-Up Period	Population	Analytical Platform	Sample Type	Endpoint	Predictive Metabolites/Pathways
Van der Kloet et al., 2012 [68]	Average of 5.5 years	52 normoalbuminuric T1DM (FinnDiane cohort subgroup)	GC-MS/LC-MS	Urine	Progression of albuminuria	Acylglycines, acylcarnitines, tryptophan metabolites/Amino acid metabolism, fatty acid beta-oxidation
Solini et al., 2016 [70]	Average of 13 years	286 T2DM patients with a median eGFR = 85 ± 20 mL/min/1.73 m^2^	GC-MS/UPLC-MS/MS	Serum and urine	Renal injury defined as eGFR < 60 mL/min/1.73 m^2^ or ACR ≥ 30 mg/g	s-C-glycosyl tryptophan, s-pseudouridine, s-N-acetylthreonine/Nucleotide and amino acid metabolism
Sas et al., 2016 [59]	Average of 5 years	Non-diabetics (control group, *n* = 28) and 26 T2DM without DKD (FIND study subgroup)	LC/MS	Urine	eGFR < 60 mL/min/1.73 m^2^ and sCr > 1.2 mg/dL (progressors)	↑ TCA cycle intermediates (succinate, citrate, fumarate, malate)/TCA cycle
Haukka et al., 2018 [71]	Average of 3.2 years	200 T1DM	GC-MS/UPLCS-MS/MS	Serum	Development of microalbuminuria	Erythritol, 3-phenylproprionate, N-trimethyl-S-aminovalerate/Gut microbiota, Pentose-phosphate pathway
Afshinnia et al., 2019 [72]	Average of 9.6 years	92 T2DM with iothalamate-based GFR ≥ 90 mL/min	LC/MS	Serum	↓ eGFR ≥ 40% compared to baseline	Long-chain acylcarnitines, TAGs/Fatty acid beta-oxidation, Lipid metabolism
Mutter et al., 2021 [73]	9 ± 5 years	2670 Τ1DM with median eGFR 81 mL/min/1.73 m^2^	NMR spectroscopy	Urine	Progression to higher albuminuria stage or progression to ESKD	2-hydroxy-isobutyrate/BCAA catabolism, Gut microbiota
Zhu et al., 2022 [74]	69 months	30 T2DM without DKD	LC/MS	Serum	Rapid ↓ eGFR (≥3 mL/min/1.73 m^2^ annually) or eGFR < 60 mL/min/1.73 m^2^ or ACR ≥ 30 mg/g	↑ L-Valine and isoleucine/BCAA metabolism
Lin et al., 2022 [75]	4 years	28 T2DM without DKD	LC/MS	Serum	eGFR < 60 mL/min/1.73 m^2^ or ACR ≥ 30 mg/g	↑ uric acid and ↓ phosphatidyloethanolamine 36:4/Purine metabolism, Lipid metabolism
Perng et al., 2025 [76]	15 years	1947 individuals with prediabetes	LC/MS	Serum	eGFR < 45 mL/min/1.73 m^2^ or end-stage renal disease, dialysis or transplantation or ACR ≥ 30 mg/g	↓ histidine and serine ↑ N-carbamoyl-beta-alanine (in the metformin treatment arm)

↑ increased concentration, ↓ decreased concentration.

## 5. Discussion

Diabetic kidney disease remains a major contributor to CKD burden and end-stage renal disease worldwide, posing profound clinical and economic challenges. Current diagnostic tools identify kidney damage only after irreversible histopathological changes have occurred. Furthermore, their prognostic accuracy is limited by the heterogeneous nature of DKD progression, where some patients experience rapid renal decline without albuminuria, while others regress or remain stable for years. These limitations underscore the urgent need for novel, predictive and non-invasive biomarkers that can identify individuals at high risk of DKD before the onset of clinically overt disease.

This review highlights how metabolomics, by capturing a snapshot of dynamic metabolic processes, offers a promising avenue for early DKD detection. A growing body of animal and human studies shows that perturbations in several key metabolic pathways, including mitochondrial energy metabolism, lipid and amino-acid metabolism, can precede albuminuria or eGFR decline in individuals with diabetes. These findings suggest that metabolomics could not only improve risk stratification but also shed light on the mechanistic underpinnings of DKD.

Notably, prospective human studies, although still limited in number, support the predictive value of specific metabolic signatures. Urinary and serum metabolites related to TCA cycle, acylcarnitines, branched-chain amino acids and uremic toxins have shown association with incident microalbuminuria and renal function decline in both type 1 and type 2 diabetes. Some studies also demonstrate that integrating metabolomics-derived scores with clinical variables significantly improves predictive accuracy for incident DKD. Further gains in predictive performance have also been observed through the integration of metabolomics with other omics approaches, underscoring the value of multi-omics approaches in capturing the multifactorial nature of DKD. These promising results highlight the added diagnostic and prognostic value that metabolomics can bring to early DKD care.

However, several challenges must be addressed. First, there is considerable heterogeneity in study design, population characteristics (e.g., diabetes type, ethnicity, comorbidities), biofluids analyzed (serum vs. urine), analytical platforms used and statistical approaches, making direct comparisons difficult. Second, while cross-sectional associations may suggest a link between certain metabolites and DKD severity, they cannot establish temporal or causal relationships. Prospective validation in larger, well-phenotyped cohorts is crucial. Third, standardization of metabolomics workflows and rigorous adjustment for potential confounders (diet, medications, etc.) are essential for reproducibility and clinical translation.

Moreover, the biological interpretation of metabolomics data remains complex. Many discriminatory metabolites participate in multiple biochemical pathways, and their levels are influenced by systemic metabolic states and not kidney function alone. Untangling disease-specific signatures from background metabolic noise requires sophisticated bioinformatics and pathway-level analyses.

Collectively, current evidence supports the utility of metabolomics as a powerful approach to uncover early biochemical perturbations preceding the clinical onset of DKD. While substantial progress has been made, studies remain exploratory and translation into clinical practice is still limited. The integration of metabolomic data with clinical and multi-omics profiles has shown promise in improving risk stratification but further validation in diverse populations, standardization of analytical pipelines and mechanistic interpretation of metabolite changes are necessary. These considerations underscore the need for coordinated efforts to move towards application, laying the groundwork for future research and clinical implementation. From a clinical perspective, while validated metabolomic panels are not yet available for routine use, the mechanistic insights provided by these studies could provide immediate practical guidance. The consistent identification of mitochondrial stress and fatty acid oxidation defects as early markers of kidney injury offers a strong biological rationale for the early initiation of renoprotective agents with known mitochondrial benefits, such as SGLT2 inhibitors and GLP-1 receptor agonists, in patients with diabetes even prior the onset of overt DKD. Additionally, the association between systemic lipotoxicity, elevated BCAAs and risk of kidney damage reinforces the need for holistic management of metabolic syndrome. Dyslipidemia and insulin resistance should be treated as direct renal risk factors to mitigate the metabolic flux reaching the kidney. Finally, institutions should encourage biobanking protocols alongside routine care. Preserving samples from high-risk patients is the only way to retrospectively validate these biomarkers once the clinical phenotype of DKD eventually emerges.

## 6. Conclusions

Despite the availability of novel therapeutic agents such as SGLT2 inhibitors, GLP-1 receptor agonists and non-steroidal mineralocorticoid receptor antagonists, the residual risk for progression to end-stage renal disease in patients with diabetic kidney disease remains unacceptably high. Furthermore, interindividual variability in treatment response highlights the heterogeneity of disease mechanisms and the need for a more nuanced understanding of DKD pathogenesis. These limitations underscore the critical importance of early diagnosis and stratified therapeutic approaches.

Metabolomics offers a promising avenue by enabling comprehensive profiling of biochemical changes that precede overt renal impairment. Identifying specific metabolite signatures associated with early disease progression facilitates novel biomarker discovery, improved risk stratification and the development of targeted interventions, supporting a precision medicine framework in DKD management.

While most existing studies remain exploratory and often lack standardization, initial findings are encouraging. Translating these insights into clinical practice, future efforts should focus on the design of large, multicenter, prospective studies of sufficient duration and well-defined clinical endpoints. These studies must implement strict standardization of pre-analytical variables (fasting status, sample processing) and utilize external validation cohorts from diverse populations to ensure that identified signatures are robust and not confounded by ethnicity or age. Furthermore, integrating metabolomics with other omics platforms and real-world clinical data will further enhance the biological interpretability and predictive accuracy of metabolomics-derived biomarkers. Additionally, efforts must be directed toward the development of robust, cost-effective, and scalable assays that can be implemented in routine clinical laboratories.

In conclusion, metabolomics holds the potential to transform the current paradigm of DKD diagnosis and management, from reactive interventions based on late-stage biomarkers to proactive, individualized care rooted in early molecular insights.

## Data Availability

No new data were created or analyzed in this study. Data sharing is not applicable to this article.

## Abbreviations

The following abbreviations are used in this manuscript:

DKD	Diabetic Kidney Disease
DM	Diabetes Mellitus
ESKD	End-stage Kidney Disease
KRT	Kidney Replacement Treatment
CVD	Cardiovascular Disease
KDIGO	Kidney Disease: Improving Global Outcomes
CKD	Chronic Kidney Disease
eGFR	Estimated Glomerular Filtration Rate
UACR	Urine Albumin-to-Creatinine Ratio
ACR	Albumin-to-Creatinine Ratio
DALYs	Disability Adjusted Life Years
AGEs	Advanced Glycation End products
NADPH	Nicotinamide Adenine Dinucleotide Phosphate
ROS	Reactive Oxygen Species
ATP	Adenosine Triphosphate
RAAS	Renin–Angiotensin–Aldosterone System
TGF	Transforming Growth Factor
CKD-EPI	Chronic Kidney Disease Epidemiology Collaboration
MDRD	Modification of Diet in Renal Disease
NAG	*N*-Acetyl-β-D-Glucosaminidase
NGAL	Neutrophil Gelatinase-Associated Lipocalin
H-FABP	Heart Fatty Acid-Binding Protein
NMR	Nuclear Magnetic Resonance
MS	Mass Spectrometry
GC	Gas Chromatography
LC	Liquid Chromatography
MDA	Multivariate Data
PCA	Principal Component Analysis
PLS-DA	Partial Least Squares-Discriminant Analysis
OPLS-DA	Orthogonal Partial Least Squares-Discriminant Analysis
TCA	Tricarboxylic Acid
GC-TOFMS	Gas-Chromatography Time-Of-Flight Mass Spectrometry
BCAA	Branched-Chain Amino Acid
UPLC-MS	Ultra-Performance Liquid Chromatography-Tandem Mass Spectrometry
FIND	Family Investigation of Nephropathy and Diabetes
DPPOS	Diabetes Prevention Program Outcomes Study
